# Some Thoughts on Mouth Hygiene*Read before the National Dental Association October 23-26, 1917, and reprinted in full from The *Journal N. D. A.*, for September, 1918.

**Published:** 1918-10

**Authors:** F. H. Skinner


### SOME THOUGHTS ON MOUTH HYGIENE*

BY F. H. SKINNER, D.D.S.

There are many procedures necessary to insure the health of the oral cavity, when an individual is not immune to dental troubles.

Let us start with the preservation and care of the temporary teeth. From the time children's teeth erupt, they should be kept free from debris and stains. Supplementary to the dentist's care, the mother or nurse should be taught how to do her part intelligently. Decay in temporary teeth usually starts in deep or imperfectly formed fissures and proximal surfaces of the molars. Cavities can be prevented in these places by covering the fissures with cement before any decay has started and by polishing the proximal surfaces with dental tape. Should these temporary molars or even their contact points be lost, the first permanent molars will erupt mesially of where they belong, the bicuspids will come in too close to the laterals, and the cuspids will be crowded out of alignment.

Mouth breathing and thumb sucking should be corrected, and adenoids should be removed. If there is not perceptible spreading of the anterior teeth at about the age of five years, a small expansion arch should be put on to stimulate the growth of the maxillary bones. A few weeks of orthodontic procedure at this age will stimulate normal growth, thus allowing the permanent teeth to erupt in good alignment and save years of regulating work at a later period in life.

As soon as the first permanent molars break through the gums, a cement of highly adhesive properties should

*Read before the National Dental Association October 23-26, 1917, and reprinted in full from *The Journal N. D. A.*, for September, 1918.

be crowded into the fissures, and as these teeth, especially, take their place in the arch, their surfaces should be kept clean, smooth and polished. From this time up to the age of fifteen or twenty years, special care should be given children's teeth, to keep the surfaces from becoming etched or roughened, thus preventing the foundation of decay at a later period in life.

### PROFESSIONAL PROMOTION OF ORAL HEALTH.

Only a small percentage of our patients have been taught to take advantage of this ideal care early enough in life to derive the benefits therefrom. Many delay their visits to the dentist until in trouble, when frequently there is much more trouble in the mouth than that which brought them in. I especially refer to the etched, roughened or undeveloped enamel surfaces, which, unless properly cared for, are bound to become cavities later on.

To put a mouth in a hygienic condition, all foreign substances must be removed from the tooth surfaces, and decay must be arrested if only by means of temporary fillings; abscesses must be cured and root canals properly filled; teeth with incurable abscesses should be extracted, and the apical region of the process curetted; bridges which can not be kept clean, and which have ill-fitting abutments, must be replaced with bridges which can be kept clean and the abutments of which do not cause irritation; fillings with over-hanging margins must be dressed down so that the restoration represents the part of the tooth lost and nothing more, *i. e.*, what nature would have formed in the ideal, as far as fissures, cusps, contact points and marginal ridges are concerned; when banded crowns are used, they should not be driven beyond the root preparation, thus eliminating overhanging edges, which are always a source of irritation to the soft tissues.

I want especially to emphasize the value of marginal ridges, because so many fillings are inserted with smooth, flat occlusal surfaces, some of which even *incline towards the contact points.* Nature forms high marginal ridges and sharp fissures. This tends to hold the teeth in position, and in mastication, it cuts and breaks up the food and forces it to the central portion of the grinding teeth. Where the surfaces are smooth, the food is only pressed out instead of being cut and macerated, and when the plane inclines mesially or distally, the force tends to separate the teeth and wedge fibrous food between the contact points.

### THE SPECIALIST

Every dentist should be a specialist in prophylaxis and pyorrhea work as well. Before placing permanent fillings and treating root canals, he should place the soft tissues in a healthy condition. For a number of years, it has been the practice of the essayist to remove all visible deposits, but before going into the deeper pyorrhea pockets, to polish the crown surfaces and build up the supporting tissues by stimulating circulation, *i. e.*, producing hyperemia, both active and passive, and when the inflammation has subsided, to remove all calcic deposits, but nothing more. In fact, better results seem to be obtained when the tooth surfaces have been cleaned and polished, and kept so from two to four weeks before any deeper scaling is done. Cases handled in this way respond more readily than when more heroic measures are employed, and the work can be done less painfully.

I believe that pyorrhea is caused directly by soft accumulations on the tooth surfaces themselves, and that the so-called serumal deposits are a result of an inflammatory condition and only one of the stages of pyorrhea. When there is inflammation in the tissues adjacent to the tooth, we usually find this deposit on that tooth, *i. e.*, this form of deposit results from the inflammation of apical abscesses, lateral abscesses or gingivitis, and in the last case, it is the so-called pyorrhea. It is my opinion that if the tooth surfaces could be kept positively free from all soft accumulations and good occlusion could be maintained, we could about eliminate gingivitis, pyorrhea and lateral abscesses.

The normal saliva is supposed to contain some tooth preserving properties, and when in actual contact with the tooth surfaces, it no doubt is beneficial, but it does not touch a plaque covered surface. In fact, acid fermentation is taking place on any tooth surface covered with soft debris. Thorough mastication of course foods, such as nuts, whole grain wheat, rye or corn bread, raw wheat, uncooked fruits, etc., minimizes the formation of plaques and adds to the immunizing properties of the saliva. When the diet is composed largely of predigested, soft foods, unless extreme care is taken, the teeth are usually covered with a fermenting mass of debris. Gingivitis, followed by osteitis and osteomyelitis, results, and retrogressive changes of the peridental tissues are sure to take place.

Gingivitis is a much more deeply seated inflammation than is ordinarily supposed, as is evidenced by congested or cyanotic areas high up on the gum tissues, with little red lines or stringers leading from the gingiva to the deeper areas. The result of this deep inflammation is the slow absorption of the process and sclerosis of the blood vessels which supply the dental and peridental tissues.

Any bone which is built up by the Haversian system is subject to constant changes. This formation is typical in the alveolar portion of the maxillary bones. When gingivitis is present, it is accompanied by more or less osteitis or osteomyelitis. Whenever there is osteomyelitis, the Haversian canals are increasing in diameter and gradually coalescing one with another at the expense of the bony structure, with the result that the bony support of the teeth gradually disappears. Apparently, changes are not confined to the alveolar process. From a microscopical examination of decalcified specimens taken from the living human jaw, it appears that it is possible for even the cementum to absorb and rebuild. Where clean tooth surfaces are maintained and the teeth are used sufficiently to produce normal health of the peridental tissues, their supporting structures are likely to remain unimpaired indefinitely.

### OCCLUSION

The average individual thirty-five or forty years of age has worn some portions of his teeth more rapidly than others, and during the process of mastication, these higher places produce an unnatural force (malocclusion). This abnormal stress lessens the resisting power of the soft tissues, and they become a ready prey to pathogenic organisms, which are always present in all mouths, even in infants three or four hours old. Nearly every case, yes, I might say every case of pyorrhea, needs some adjusting of the occlusion by grinding. Frequently, a loose tooth will tighten within forty-eight hours after the high places have been ground to produce normal occlusion. Often, an elongated loose tooth, when not entirely detached, can be saved by being depressed well into the process. Depressing is an orthodontic procedure, and stimulates the growth of bone.

### HOME CARE OF THE MOUTH

When a dentist has put a patient's mouth in a hygienic condition, he has done less than half his duty, because the same conditions which previously existed will reproduce the disease. The after care is just as important as the work of producing health in a mouth. Patients are frequently dismissed with the idea that, after the tooth surfaces have been cleaned and polished, they are immune to dental troubles for the rest of their natural lives. This is a great mistake, because within three or four days, accumulations will begin to gather on even the most highly polished tooth surfaces. An appointment should be made for prophylaxis treatment within ten days after a case has been completed.

Let a patient see the accumulations which have gathered in such a short time, as evidenced by a staining solution. Show him how much debris he could have removed by the proper use of a tooth brush and a good dentifrice. Then apply the disclosing solution a second time. In all probability, it will still show a narrow line of debris next to the gums, and the proximal surfaces will be far from clean. Teach him how to use wooden points to remove the debris next to the gum margin and dental tape to clean the proximal surfaces. Cotton rolls or cheese cloth napkins are very efficient for cleaning any surface they will reach, and cotton rolls are especially beneficial for massaging the gums, because they stimulate circulation without doing any injury to the soft tissues, nor will they injure the tooth surfaces. At this sitting, work on the teeth until the disclosing solution stains nothing more, then let the patient see that his teeth are clean, and impress upon him the necessity for keeping them so.

He may be dismissed for thirty days. After this, the period for prophylaxis treatments is determined by the length of time a patient is able to keep his teeth clean. It sometimes takes a year or two for patients to learn how to maintain a healthy condition, but persistence on our part will win out in a majority of cases, and the satisfactory results will justify the work. I know of nothing more pleasing to a patient than to let him see and feel his mouth in a clean, wholesome condition.

Maintaining healthy teeth and gum tissues for our patients is a practice builder, and I hope the time will come when the practice of oral prophylaxis shall be the regular routine work of every dental office.

